# Preimplantation factor modulates oligodendrocytes by *H19*-induced demethylation of *NCOR2*

**DOI:** 10.1172/jci.insight.132335

**Published:** 2021-10-22

**Authors:** Marialuigia Spinelli, Celiné Boucard, Sara Ornaghi, Andreina Schoeberlein, Keller Irene, Daniel Coman, Fahmeed Hyder, Longbo Zhang, Valérie Haesler, Angelique Bordey, Eytan Barnea, Michael Paidas, Daniel Surbek, Martin Mueller

**Affiliations:** 1 Department of Obstetrics and Gynecology and Department of Biomedical Research, University Hospital Bern, University of Bern, Bern, Switzerland.; 2 Department of Obstetrics, Gynecology, and Reproductive Sciences, Yale University School of Medicine, New Haven, Connecticut, USA.; 3 Department for Biomedical Research and Swiss Institute of Bioinformatics, University of Bern, Bern, Switzerland.; 4 Department of Radiology and Biomedical Imaging,; 5 Department of Biomedical Engineering,; 6 Department of Neurosurgery, and Department of Cellular & Molecular Physiology, Yale University School of Medicine, New Haven, Connecticut, USA.; 7 Department of Research, BioIncept LLC, New York, New York, USA.

**Keywords:** Neuroscience, Therapeutics, Drug therapy, Neurodevelopment

## Abstract

Failed or altered gliogenesis is a major characteristic of diffuse white matter injury in survivors of premature birth. The developmentally regulated long noncoding RNA (lncRNA) *H19* inhibits *S*-adenosylhomocysteine hydrolase (SAHH) and contributes to methylation of diverse cellular components, such as DNA, RNA, proteins, lipids, and neurotransmitters. We showed that the pregnancy-derived synthetic PreImplantation Factor (sPIF) induces expression of the nuclear receptor corepressor 2 (NCOR2) via *H19*/SAHH-mediated DNA demethylation. In turn, NCOR2 affects oligodendrocyte differentiation markers. Accordingly, after hypoxic-ischemic brain injury in rodents, myelin protection and oligodendrocytes’ fate are in part modulated by sPIF and *H19*. Our results revealed an unexpected mechanism of the *H19*/SAHH axis underlying myelin preservation during brain recovery and its use in treating neurodegenerative diseases can be envisioned.

## Introduction

Advances in obstetric and neonatal care have successfully increased survival rates of very premature infants, but the cost is the increase in preterm-specific brain injuries (1). The majority of prematurity survivors face long-term neurodevelopmental disabilities, including cerebral palsy, motor deficits, or/and cognitive impairments that persist at least to young adulthood (2). The hallmark of this injury is inflammation and altered gliogenesis with resulting diffuse white matter damage (3, 4). Neurogenesis and gliogenesis are precise spatially and temporally regulated processes, where neurons are generated in an inside-out fashion first, followed by the production of astrocytes and/or oligodendrocytes (5, 6). In line with this notion, the peak period of diffuse white matter injury occurrence (24–32 weeks of gestation) coincides with the highest presence of immature oligodendrocytes in the white matter (7, 8). Thus, targeting and modulating oligodendrocytes’ fate in an immature brain after injury is an attractive strategy but has remained elusive (9, 10).

Gene methylation (5-methyl-cytosine) plays a critical role in mammalian development and is catalyzed by the joint action of 3 *S*-adenosylmethionine–dependent DNA methyltransferases (DNMTs) (11). DNMTs and specific methylation/demethylation programs determine cell specificity and fate during neuro- and gliogenesis (12, 13). The specific interaction between DNMTs and long noncoding RNAs (lncRNAs) has emerged as an integral part of corticogenesis (14). The developmentally regulated imprinted lncRNA *H19* (called *H19* herewith) is of special interest. *H19* is highly expressed during corticogenesis and plays important roles in embryo development and growth control (15). Besides its presence in embryonal tissues (16), *H19* is detected in a subset of postnatal and adult tissues, including the skeletal muscle (17, 18) and endometrium (19). *H19* encodes a polyadenylated lncRNA of approximately 2.6 kb, which is predominantly cytoplasmic, with a minor fraction also found in the nucleus (20). Importantly, *H19* binds to *S*-adenosylhomocysteine hydrolase (SAHH) and inhibits its function both in vivo and in vitro (16, 21). SAHH is the major mammalian enzyme capable of hydrolyzing *S*-adenosylhomocysteine (SAH) to homocysteine and adenosine and therefore SAH, as a potent feedback inhibitor of *S*-adenosylmethionine–dependent methyltransferases, binds to DNMTs and prevents DNA methylation. The specific role of *H19* during gliogenesis is not known.

PreImplantation Factor (PIF) is an evolutionarily conserved embryo-derived peptide (MVRIKPGSANKPSDD) detectable in maternal circulation of viable pregnancies, and similar to *H19*, it is expressed by the embryo/fetus and placenta (15, 22–26). Interestingly, besides PIF’s effects on maternal toleration and embryo implantation (27), a synthetic PIF analog (sPIF) was able to reverse and prevent paralysis and restore myelination through inhibiting neuroinflammation in murine models of experimental autoimmune encephalomyelitis (28, 29). The neuroprotective property of sPIF was further underscored by its ability to mitigate microglial activation and neuronal loss while promoting neuronal migration in murine models of perinatal brain injury (30, 31). These neuroprotective effects, at least in part, were due to altered miRNA *let-7* synthesis (30). Notably, *let-7* regulates radial migration of neurons, and its bioavailability is affected by *H19* (32, 33). Recently, sPIF was reported to modulate myoblast differentiation through the *H19*/mir-675/*let-7* axis (17), and therefore we speculated that the sPIF/*H19* axis may be involved in oligodendrocyte formation as well.

Given the emerging importance of epigenetic regulation during corticogenesis (34) and DNA global methylation changes induced by *H19* (21), we hypothesized that sPIF/*H19* affects oligodendrocytes’ fate by *S*-adenosylmethionine–dependent DNA methylation. In the current study, we found that sPIF induced expression of the nuclear receptor corepressor 2 (NCOR2) via *H19*/SAHH-mediated DNA demethylation. In turn, NCOR2 contributes to oligodendrocyte differentiation. We provide in vivo evidence that oligodendrocyte differentiation and myelin preservation are in part modulated by the sPIF/*H19* pathway. Our results revealed an unexpected mechanism of the *H19*/SAHH axis in myelin preservation during brain recovery, bearing the potential that the discovered *H19*/SAHH axis can be used as target in drug discovery programs.

## Results

### sPIF induces oligodendrocyte differentiation markers in an H19-dependent manner.

**Figure 1:**
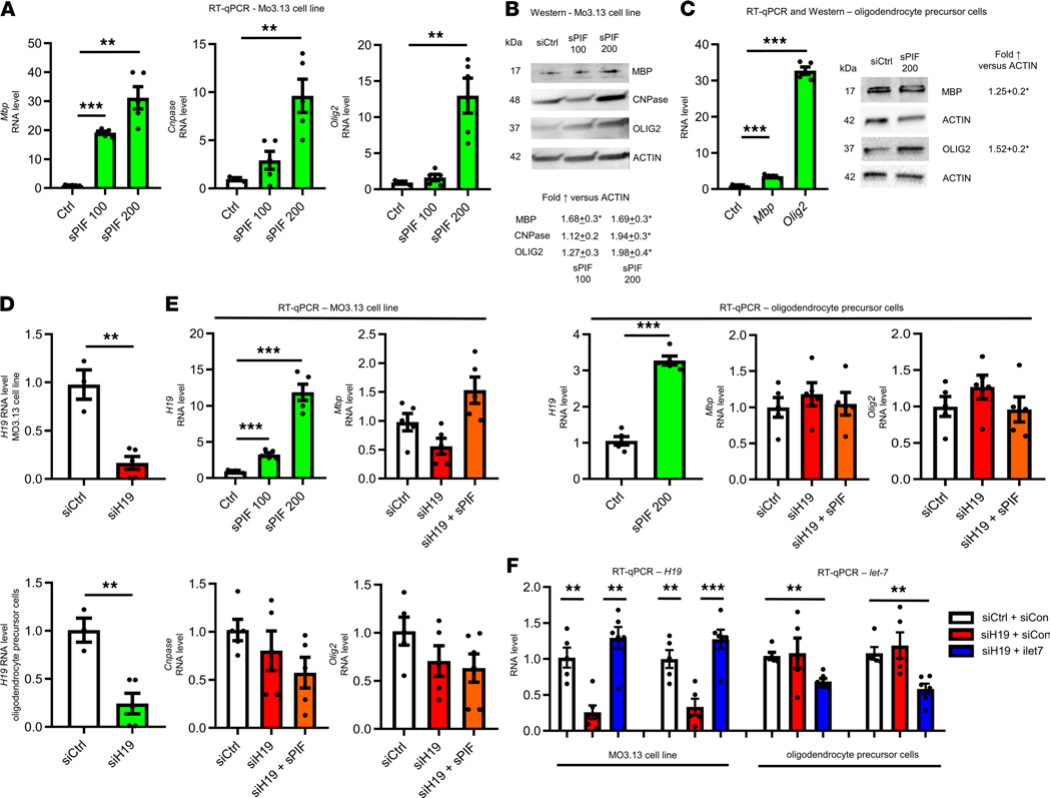
sPIF induces oligodendrocyte differentiation markers in an *H19*-dependent manner. Hybrid cell line (MO3.13) that expresses phenotypic characteristics of primary oligodendrocytes in an immature developmental stage and rat oligodendrocyte precursor cells were incubated with sPIF (100 and/or 200 nM or control — Ctrl). RNAs and proteins were extracted 48 hours later, and levels were determined by qRT-PCR (**A** and **C**) and Western blots (**B** and **C**: representative Western blots). (**D** and **E**) Cells were transfected with control siRNA (siCtrl) or siRNA specific for *H19* (siH19) or incubated with sPIF at 100 and/or 200 nM or Ctrl. RNAs were harvested 48 hours after transfection and analyzed by qRT-PCR. (**F**) Cells were transfected with control siRNA (siCtrl) and/or control miRNA (siCon) and siRNA specific for *H19* (siH19) or *let-7* inhibitor (iLet7), and RNAs were harvested 48 hours after transfection and analyzed by qRT-PCR. Results are presented as mean ± SEM and are representative of at least 3 independent experiments. Single comparisons with Ctrl were made using a 2-tailed Student’s *t* test or Mann-Whitney test with Bonferroni’s correction. **P < 0.005; ***P < 0.0005. The levels of Ctrl and siCtrl were arbitrarily set to 1. Protein levels are presented after normalization to actin. Each experiment was conducted at least 3 times.

To test whether sPIF/*H19* modulates oligodendrocyte differentiation markers, we used an immortal human-human hybrid cell line (MO3.13) that expresses phenotypic characteristics of primary oligodendrocytes in an immature developmental stage and rat oligodendrocyte precursor cells (35). We treated cells with sPIF or scrambled PIF peptide (control) and detected increased markers of myelinating, early differentiating oligodendrocytes, and oligodendrocyte progenitor cells, namely myelin basic protein, 2′,3′-cyclic nucleotide 3′-phosphodiesterase (CNPase), and oligodendrocyte transcription factor 2 (Olig2), at mRNA and protein levels (Figure 1, A–C). Given the importance of *H19* and PIF during embryogenesis (15, 22–26), we tested sPIF in the presence of *H19*-specific siRNA (siH19) next (Figure 1, D and E) (21). In line with previous results (17), sPIF increased the expression of *H19* significantly (Figure 1E), while losing the ability to induce oligodendrocyte differentiation markers in the presence of siH19 (Figure 1, D and E). Together, our results support the notion that sPIF induces oligodendrocyte differentiation markers in an *H19*-dependent manner.

*H19* acts as a molecular sponge for *let-7*, contributing to differentiation of muscle (17, 18) or endometrium (19). We therefore aimed to test the role of sPIF and *let-7* next (30). For this purpose, we used a *let-7*–specific inhibitor (iLet7) concomitant with *H19* knockdown (18). Notably, iLet7 is a chemically modified, single-stranded nucleic acid that specifically binds to *let-7* molecules and inhibits endogenous *let-7* (12). Thus, cells transfected with siH19 plus iLet7 should mimic in part sPIF’s effects (reduced *let-7* bioavailability) (17, 30). Surprisingly, siH19 plus iLet7 transfection did not increase oligodendrocyte differentiation markers (Figure 1F and Supplemental Figure 1; supplemental material available online with this article; https://doi.org/10.1172/jci.insight.132335DS1), which suggests that the observed effects (Figure 1, A–E) are *let-7* independent. These results further support the notion that sPIF induces markers of different oligodendrocyte differentiation stages in an *H19*-dependent manner. Next, we thought to uncover the exact *H19*-mediated mechanism.

### sPIF and H19 induce DNA methylation changes in oligodendrocytes.

**Figure 2:**
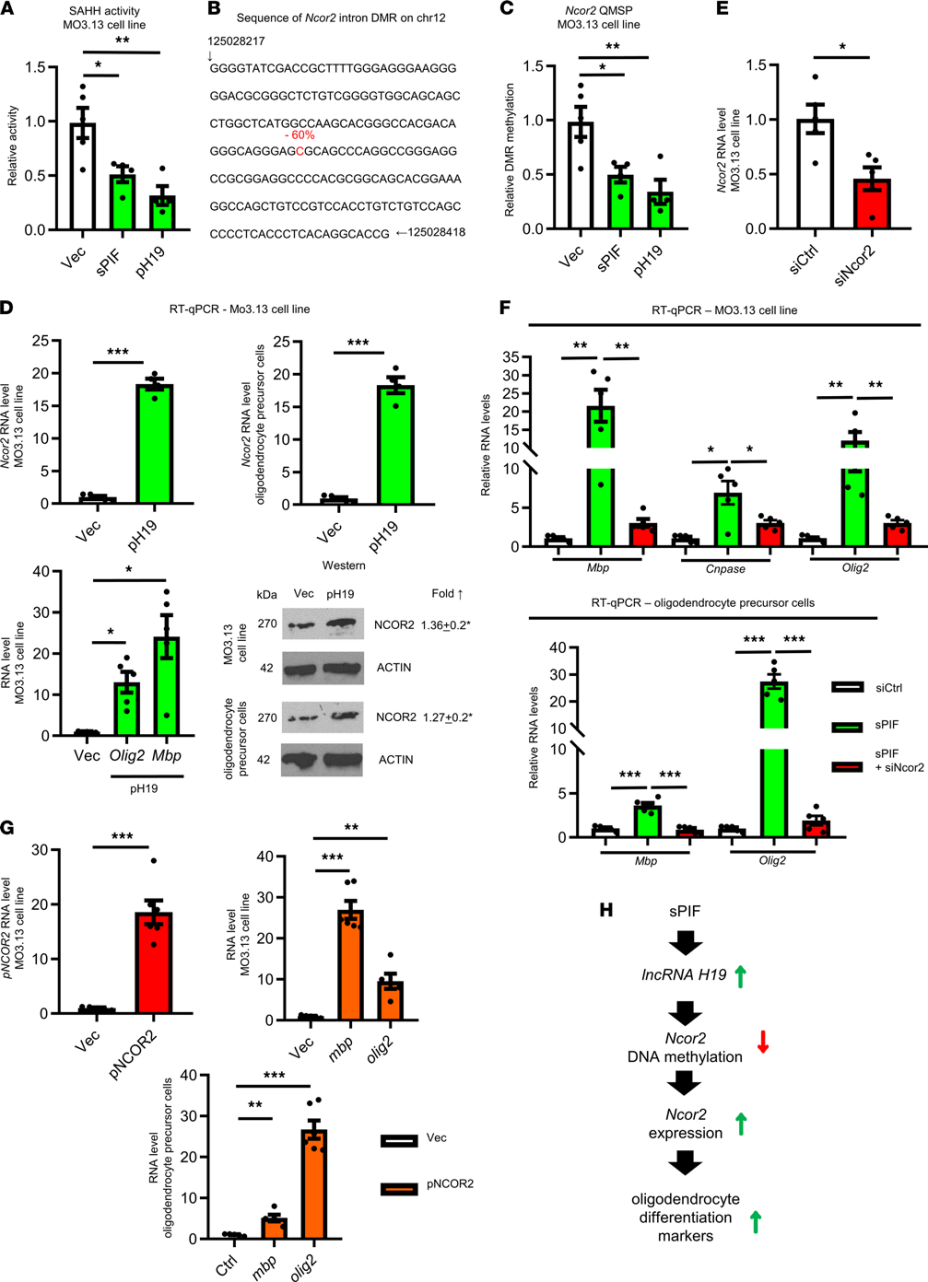
sPIF/*H19* modulates oligodendrocyte differentiation markers by demethylation of *Ncor2*. MO3.13 cells were transfected with vector (Vec), *H19*-containing plasmid (pH19), or sPIF (200 nM) and after 48 hours analyzed. (**A**) We measured *S*-adenosylhomocysteine hydrolase (SAHH) and (**B**) performed global genome methylation analysis. One specific sequence of the differentially methylated region–amplified (DMR-amplified) *Ncor2* intron region with reduced methylated cytosine is highlighted in red. Numbers at the beginning and end of the sequence mark positions of the indicated nucleotides in the chromosome. The number above in red indicates the percentage of methylation in Vec compared with treatment (pH19 or sPIF; *n* = 3 each group). (**C**) We confirmed the specific hypomethylation site (as indicated in **B**) using quantitative methylation-specific PCR (QMSP) analysis. We measured *Ncor2* expression at mRNA and protein levels (representative Western blots) after pH19 transfection (**D**). We confirmed *Olig2* and *Mbp* induction using pH19 (**D**) and *Ncor2* silencing in (**E**). (**F**) We measured mature and immature oligodendrocyte expression markers after sPIF (200 nM) treatment and specific siNCOR2 transfection. (**G**) These markers were measured after Ncor2 overexpression (**G**) as well. (**H**) Proposed model of sPIF to modulate oligodendrocyte fate by *H19*-induced hypomethylation of *Ncor2*. Single comparisons to Ctrl were made using a 2-tailed Student’s *t* test or Mann-Whitney test with Bonferroni’s correction. *P < 0.025; **P < 0.005; ***P < 0.0005. The levels of Ctrl and siCtrl were arbitrarily set to 1. Protein levels are presented after normalization to actin. Each experiment was conducted at least 3 times.

To dissect the role of sPIF/*H19* during oligodendrocyte differentiation, we treated cells with sPIF, human expression plasmid H19 (pH19), or control empty vector (Vec) (16) and measured SAHH activity. In line with the notion that *H19* inhibits SAHH activity (16, 21), we detected a significant decrease after sPIF treatment or pH19 transfection (Figure 2A). Given that SAHH modulates DNMT-mediated DNA methylation (16, 21) and as a proof of principle, we performed global DNA methylation studies to identify downstream gene candidates regulating oligodendrocyte differentiation. We used pH19, sPIF, and Vec in an MO3.13 cell line (*n* = 3 each group) again and performed genome-scale DNA methylation mapping using the platform of an improved version of reduced representation bisulfite sequencing (RRBS). This platform offers the identification and analysis of differentially methylated regions between samples at single-nucleotide resolution. As expected, both sPIF and pH19 induced extensive genome-wide methylation pattern changes relative to Vec (Supplemental Table 1). We observed some genes showing increased methylation, others showing decreased methylation, and a third group with no significant changes. These changes are expected because methylation status of a given cytosine residue within a gene locus is determined not only by the activity of methyltransferases but also by other associated epigenetic marks known to influence the accessibility of methyltransferases (21). Further, not all methyltransferases display an equal sensitivity to SAHH inhibition (36). Thus, *H19* upregulation, via reduced SAHH activity, is expected to produce these different outcomes. Together, our results provide evidence that sPIF and *H19* induce global methylation changes, and these are in part SAHH mediated. Next, we aimed to uncover the gene candidates regulating oligodendrocyte differentiation.

### sPIF and H19 induce demethylation of Ncor2.

In search of gene candidates regulating oligodendrocyte differentiation, we detected that both sPIF and pH19 induced significant hypomethylation in the intron regions of NCOR2 (*Ncor2*, also known as *Smrt*) (Supplemental Figure 2). NCOR2 is an intrinsically disordered hub protein, which binds and assists transcription factors and chromatin-modifying enzymes (37). This is of interest since NCOR2 defends against the ability to initiate a differentiation program along a neuronal pathway in neural stem cells and modulates the astroglial differentiation in concert with NCOR1 (38). To confirm the detected hypomethylation (60% sPIF and pH19 versus Vec) of the predicted *Ncor2* intron region (Figure 2B), we performed bisulfite conversion followed by quantitative methylation-specific PCR analysis using primers that specifically amplify the single differentially methylated region of *Ncor2*. We detected reduced methylation of the predicted CpG site (Figure 2C), in line with global methylation analysis (Figure 2B). Since the hypomethylation of intron regions may enhance the expression of the downstream gene, we suspected that *H19*, by inhibiting SAHH and the methyltransferases, should ultimately stimulate the expression of *Ncor2*. Therefore, we transfected cells with pH19 again and indeed, pH19 induced *Ncor2* expression at mRNA and protein levels (Figure 2D). Together, these results strongly suggest that the sPIF/*H19* axis regulates, SAHH dependently, the methylation and increase of NCOR2*,* which ultimately should trigger the oligodendrocyte differentiation cascade.

To validate the hypothesis that oligodendrocyte differentiation is controlled by sPIF/*H19*/NCOR2, we used sPIF treatment in the presence of NCOR2-specific siRNA (siNCOR2) in cells again (Figure 2E). Indeed, the presence of siNCOR2 abolished sPIF’s ability to induce oligodendrocyte differentiation markers (Figure 2F), and NCOR2 overexpression resulted in similar effects (Figure 2G). Taken together, these results suggest that we uncovered a potentially novel signaling pathway that modulates oligodendrocyte differentiation markers (Figure 2H). We next decided to test the relevance of this hypothesis in vivo.

### sPIF and H19 induce differentiation of cells from the subventricular zone.

**Figure 3:**
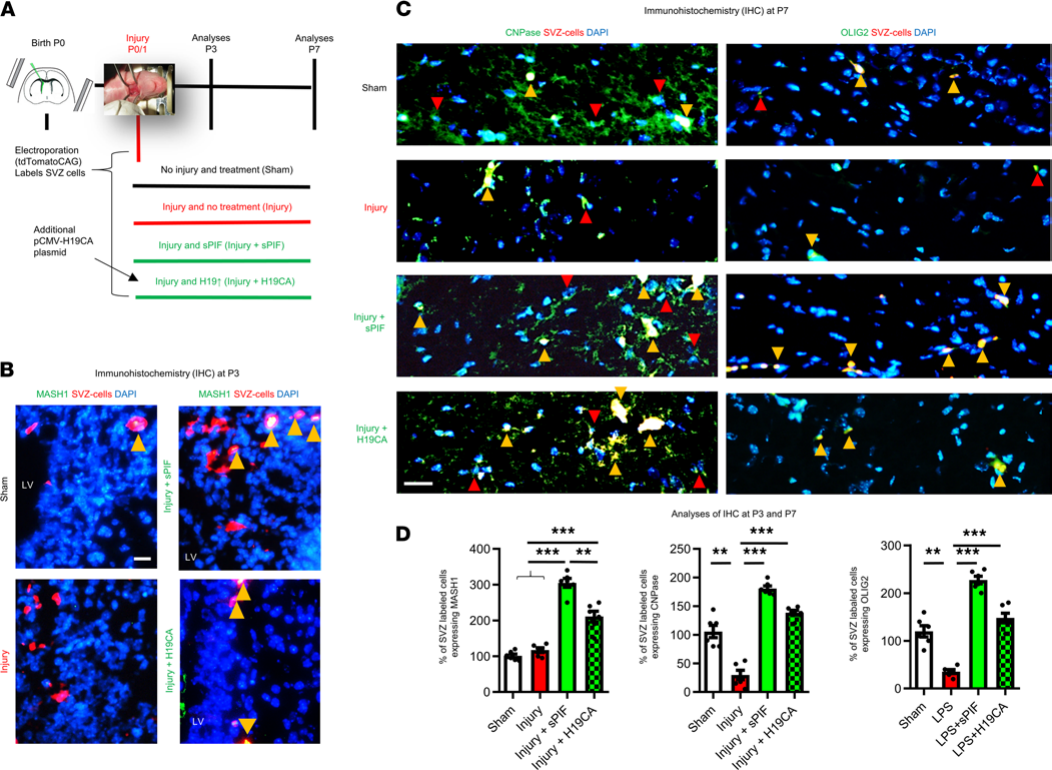
sPIF/*H19* impacts oligodendrocyte differentiation in vivo. (**A**) Experimental protocol indicating time points of electroporation, injury, treatment groups, and analyses. (**B**) Representative immunostainings of lateral ventricle (LV), cingulum, and partially caudate putamen at P3. The SVZ-labeled cells are red and SVZ/MASH1^+^ cells (differentiation marker) are indicated by yellow arrowheads (*n* = 3 each group). (**C**) Representative immunostainings of corpus callosum and deep cortical layers on P7. The SVZ/CNPase^+^ (left panels) or SVZ/OLIG2^+^ cells (right panels) representing oligodendrocyte precursors derived from SVZ-labeled cells are indicated by yellow arrowheads, and red arrowheads indicate examples of CNPase^+^ or OLIG2^+^ cells (*n* = 5 each group). (**D**) Quantification of immunostaining on P3 and P7 using percentage of SVZ-labeled cells coexpressing specific marker (*n* = 8 each group). SVZ, subventricular zone. Scale bars: 50 μm. All results are presented as mean ± SEM. **P < 0.01; ***P < 0.001 by 1-way repeated-measures ANOVA followed by Bonferroni’s multiple-comparison test.

We decided to test the role of sPIF and *H19* on oligodendrocyte maturation in vivo using a murine model of immature brain injury next. This model consisted of LPS induction followed by ligation of 1 carotid artery and a hypoxic-ischemic period at P0/1 (Figure 3A). We chose this model because injury at this time point in mice corresponds in terms of white matter development to very preterm human infants, who are highly susceptible to white matter damage (30, 31, 39–41). To follow up on the differentiation capacity of oligodendrocytes, we used the injury model in combination with electroporation. Electroporation allows the selective labeling and manipulation of radial glia cells from the subventricular zone (SVZ) and therefore follow-up on glial cells’ progeny (42). We electroporated all animals with the plasmid tdTomatoCAG that encodes the red fluorescent protein tdTomato to label neural progenitor cells and their progeny, and in the H19^CA^ group, we used an active plasmid encoding *H19* (pCMV-H19^CA^) to increase *H19* activity in the electroporated cells. In the sPIF treatment group, we s.c. injected sPIF (0.75 mg/kg birth weight twice daily starting at injury time point). We analyzed the brains at P3 or P7. At P3, both sPIF and H19^CA^ increased the differentiation (MASH-1 marker) of cells labeled in the SVZ (Figure 3, B and D; compare sPIF and H19^CA^ with injury). Notably, as a result of asymmetric division, these cells may generate transit-amplifying cells, which in turn have the potential to generate not only neurons in the olfactory bulb but oligodendrocytes in the white matter and cortex (5, 8). Therefore, we aimed to evaluate the generation of an oligodendrocyte progenitor in P7 cells next.

### sPIF and H19 induce oligodendrocyte differentiation and preserve myelin after injury.

To understand the specific effect of sPIF and H19^CA^ during gliogenesis after injury, we used IHC and diffusion tensor imaging (DTI) on P7. First, we evaluated established precursor markers of oligodendrocytes and focused on the corpus callosum and deep cortical layers (43, 44). Notably, these are the areas of expected injury in an immature brain (30, 31, 39). We detected fewer SVZ-labeled cells differentiating to CNPase and OLIG2 after injury (Figure 3, C and D; compare injury and sham groups), which is in agreement with increased susceptibility of oligodendrocyte precursors to an insult during gliogenesis (8, 45). Furthermore, both sPIF and H19^CA^ increased the number of SVZ-labeled cells expressing immature oligodendrocyte markers, suggesting an increased generation of oligodendrocyte precursors (Figure 3, C and D; compare green and red bars). These results suggest that both sPIF and H19^CA^ modulate SVZ-derived glial cells after injury.

**Figure 4:**
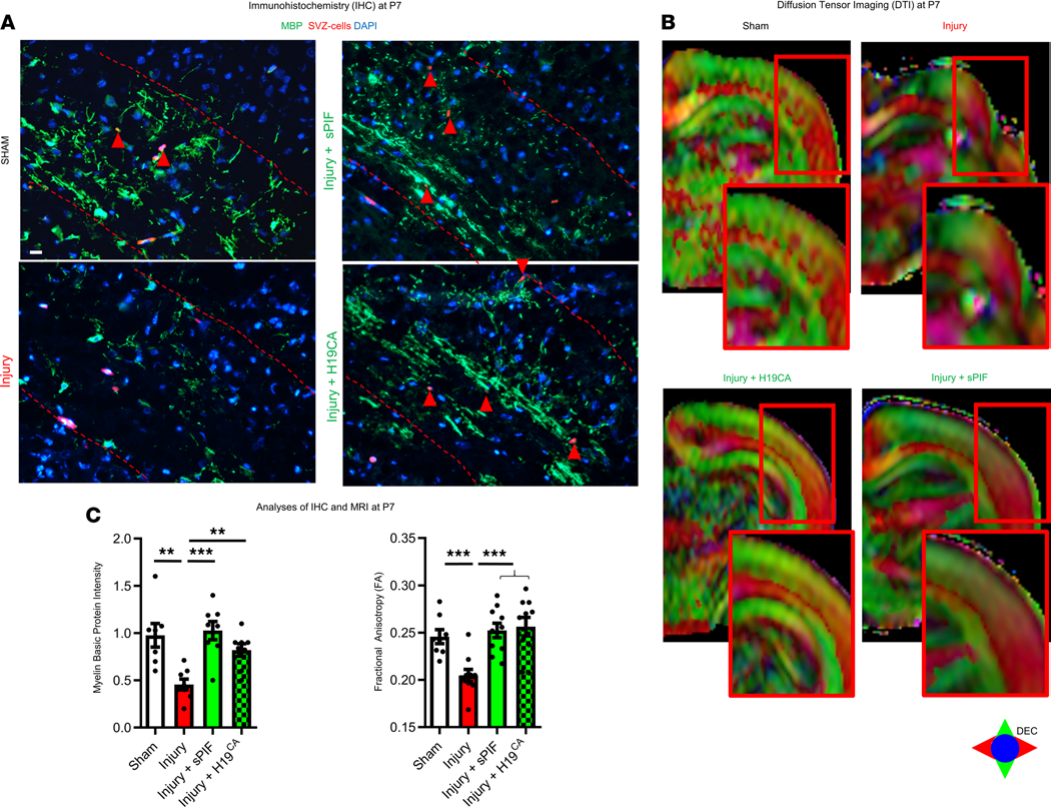
sPIF/*H19* modulates myelination in vivo. (**A**) Representative immunostaining of myelin basic protein from corpus callosum and deep cortical layers and (**B**) images from MRI (fractional anisotropy using diffusion tensor imaging). Red-edged insets represent magnification of the analyzed region of interest. (**C**) Quantification of white matter loss using immunostaining and fractional anisotropy. Scale bars: 50 μm. All results are presented as mean ± SEM. **P < 0.01; ***P < 0.001 by 1-way repeated-measures ANOVA followed by Bonferroni’s multiple-comparison test.

Given the impact of sPIF and *H19* on oligodendrocyte differentiation, we aimed to test the impact on myelination next. We used myelin basic protein IHC and DTI on P7. Notably, DTI and especially fractional anisotropy are established MRI techniques to measure directional water diffusion in the nervous system (46). The fractional anisotropy scale ranges from 0 (isotropic) to 1 (anisotropic) and reflects the ratio of the water diffusivity. Lower fractional anisotropy correlates with myelin loss (47), and the association between DTI measures and neuromotor performance is well defined (48, 49). We detected impaired myelin basic protein intensity (Figure 4, A and C; compare injury and sham groups) and reduced fractional anisotropy of the corpus callosum in injured animals (Figure 4, B and C; compare injury and sham groups). Both sPIF and H19^CA^ modulated these deficits significantly (Figure 4, A–C; compare green and red bars). Although we detected only a few SVZ-labeled cells expressing myelin basic protein (Figure 4A; red arrowheads in the panels), we provide evidence that sPIF modulates oligodendrocyte and myelin development after injury in part by *H19* regulation, underlining the role of SVZ-derived cells during brain maturation (8). Furthermore, the results provide evidence that the sPIF/*H19*/SAHH/NCOR2 regulatory axis (Figure 2H) is active in vivo and affects oligodendrocyte maturation.

## Discussion

We found that in vitro sPIF affected oligodendrocyte differentiation markers by increasing expression of *H19*, leading to decreased SAHH activity and global DNA methylation changes. One of the demethylated genes was *Ncor2*, which modulates gliogenesis. We also demonstrated that immediate s.c. supplementation of sPIF after immature brain injury in mice led to significant oligodendrocyte modulation, resulting in part in myelin preservation. Finally, sPIF-induced neuroprotection was in part due to *H19*-induced oligodendrocyte modulation.

Curiously, the adult brain expresses very low levels of *H19* (50), and in stroke *H19* promotes neuroinflammation (51). This is surprising because we demonstrated here that sPIF increased *H19*, and the inhibition of neuroinflammation in adult brains was previously reported (28, 29). However, we need to consider differences in the spatiotemporal expression and transcriptional alteration of noncoding RNAs during brain development (52). Additionally, the influence of additional methylation mechanisms such as DNA hydroxymethylation (5-hydroxymethylcytosine), which are prominent during postnatal development, need to be accounted for (53, 54). It is conceivable that cofactors of sPIF likely act in concert to elicit downstream effects that are specific to sPIF but different depending on the maturation stage of the brain. Identification of PIF-interacting cofactors is currently underway as in the case of insulin-degrading enzyme in Alzheimer disease (55). The exact mechanisms of how sPIF interacts with the cell to induce differentiation (upstream of *H19*) has not been addressed yet but is beyond the scope of this manuscript.

Our study provides evidence that ambient cell-extrinsic cues affect maturation fate of oligodendrocytes in concert with a complex of cell-intrinsic epigenetic mechanisms (34). These include the spatiotemporal regulation of cells during astrogliogenesis (5, 8). Although neurons are the first cell type arising from transit-amplifying cells during development, different signals trigger DNA demethylation and may regulate the differentiation plasticity and therefore fate of these cells. However, we speculate that the regulation of the astroglial pathway depends on proper gene dosage of both NCOR1 and NCOR2, while differentiation along the neuronal pathway appears to be regulated by loss of NCOR2 alone (38). In line with this, NCOR2 mRNA is primarily expressed in the ventricular zone region, where multipotent neural precursors reside, whereas the expression of NCOR1 is more broadly distributed throughout the dorsal cortex.

We are aware that the role of NCOR in oligodendrogenesis is controversial. The absence of NCOR promoted oligodendrocyte-associated gene expression (56), while histone acetylation modifier HDAC3 requires NCOR complex to promote deacetylation, leading to the repression of neuronal and increase of glial marker expression (57). Specifically, within the glial lineage, NCOR downregulated astrogenesis via JAK/STAT inhibition and promoted oligodendrogenesis via deacetylation and inactivation of the astrogliogenesis-promoting factor STAT3 (58). We acknowledge that the specific NCOR1-NCOR2 interactions were not evaluated here and are beyond the scope of this manuscript. Additionally, we acknowledge that using immortal cells and murine oligodendrocyte precursor cells, which have not been differentiated to mature oligodendrocytes, has certain limitations. Also, the exact postnatal SVZ cell fate after injury was not studied.

We propose that the epigenetic regulatory pathway in Figure 2H may underlie the DNA methylation dynamics associated with development and diseases, such as the regulation of neuronal fate program (38). Given the importance of PIF during embryonal/fetal development (23, 59) and the high expression of *H19* during this period (15, 16), the question whether the sPIF/*H19* axis is active beyond oligodendrocyte modulation and the impact on preterm birth or preeclampsia remains to be investigated (26). Further investigation is necessary to evaluate the exact mechanisms and fate of SVZ-derived cells after injury (8). Our results provide evidence that sPIF modulates the fate of SVZ-derived cells supporting neuroprotection, but clinical trials in immature infants warrant deeper understanding. sPIF has a short half-life in circulation (60) and despite this rapid clearance, we and others observed neuroprotection (28–31). This would indicate that the circulating PIF concentration is not a good indicator of the local concentration of sPIF at its site of action and the need to use higher concentrations of sPIF in cell culture experiments (30). This would lead to the question of proper dosage during pregnancy or in the neonate. Given sPIF’s safety profile, the FDA’s fast-track approval for clinical trials in autoimmune hepatitis (60), and the pregnancy-derived origin (27), the acceptance and use of sPIF in pregnancy or the neonate can be envisioned. A phase I safety study in preterm infants at risk of brain injury is currently in preparation.

## Methods

### Induction of immature brain injury and electroporation.

Four pregnant SD-1 mice were purchased from Charles River Laboratories, and 32 neonatal animals were randomly divided into 4 groups (Sham, Injury, and Injury+sPIF, Injury+H19^CA^; *n* = 8 in each group). All animals were electroporated at P0 using a tdTomato-encoding plasmid (tdTomatoCAG) to label neural progenitor cells from the SVZ and their progeny. Animals in the Injury+H19^CA^ group additionally received an active plasmid encoding *H19* (pCMV-H19^CA^). Electroporation was performed as we previously described (61). Plasmids (1–2 mg/mL) were diluted in PBS containing 0.1% fast green as a tracer. A total of 0.5–1 mL of plasmid solution was injected into the lateral ventricles of cold-anesthetized neonatal pups using a pulled glass pipette (diameter <50 mm). Five square pulses of 50 ms duration with 950 ms intervals at 100 V were applied using a pulse ECM830 BTX generator and tweezers-type electrodes (model 520; BTX) placed on the heads of P0 pups. We used the following plasmids: tdTomato-RFP (61), pCAG-RFP (61); active plasmid encoding *H19* (pCMV-H19^CA^; see Supplemental Methods); and vector (Ambion, AM4636) as control. We induced injury as previously reported (30, 31, 39). Briefly, animals received LPS (0.1 mg/kg BW, i.p. at P0/1) and 12 hours later, 1 common carotid artery was dissected and cauterized using a stereomicroscope. Animals were subjected to 40 minutes of hypoxia (8% O_2_/92% N_2_), resulting in a unilateral white matter injury to the corpus callosum and deep cortical layers, mimicking injury in survivors of premature birth (30, 31, 39). Starting after induction of injury, we performed the following treatments: the Injury and Injury+H19^CA^ groups received PBS; the Injury+sPIF group received sPIF (0.75 mg/kg birth weight s.c. twice daily until tissue harvesting). The Sham group consisting of sham-operated animals (0.9% NaCl instead of LPS, exposure of carotid artery without ligation, no hypoxia) received PBS and was the control group. We harvested the brains on P3 (*n* = 3 each group) and P7 (*n* = 5 each group) for further analyses.

### Cultured cells and treatments.

We obtained rat oligodendrocyte precursor cells (ROPCs) from ScienCell Research Laboratories. These cells were isolated from P2 rat brain and characterized by immunofluorescence with antibodies specific for A2B5 and O1. ROPCs were cryopreserved and delivered frozen. Upon delivery, we seeded and grew cells according to the manufacturer’s protocol. Briefly, cells were seeded at a density of 10,000 or more cells/cm^2^ in 6-well chamber slides in oligodendrocyte precursor cell medium supplemented with PBS and 1× penicillin/streptomycin. The chambers were coated with poly-L-lysine according to the manufacturer’s recommendations prior to the seeding of ROPCs. We used oligodendrocyte precursor cell differentiation medium (OPCDM, 1631). We replenished the medium after 24 hours and then exposed cells to PIF (200 nM) for 72 hours. The sPIF peptide was replenished every 24 hours in fresh serum-free media until RNA and protein extraction for further analyses. We repeated each experiment at least 3 times.

MO3-13 cells (CELLution Biosystems Inc.) are an immortal human-human hybrid cell line with the phenotypic characteristics of primary oligodendrocytes. Cell were grown in DMEM (GIBCO Invitrogen), containing 4.5 g/L glucose (GIBCO), supplemented with 10% FBS (Sigma-Aldrich), 100 U/mL penicillin, and 100 μg/mL streptomycin. The cells were kept in a 5% CO_2_ and 95% air atmosphere at 37°C. Trypsin-EDTA 0.25% (Thermo Fisher Scientific) was used to detach cells from culture plates. For treatments, cells were seeded in 24-well plates in regular growth media at a density of approximately 1.5 × 10^5^ per well. The next day, media were replaced with serum-free media containing sPIF or scrambled PIF (scrPIF) (see supplemental material) at the final concentration of 100 nM or 200 nM, followed by incubation for 48 hours. The sPIF or scrPIF peptide was replenished every 24 hours in fresh serum-free media until RNA and protein extraction for further analyses. For overexpression and silencing experiments, cells were transfected in a 24-well plate. To prepare siRNA transfection solution for each well, 30 pmol of control siRNA (siCon) or siH19 was mixed with 100 μL OPTI-MEM by gentle pipetting. In parallel, 3 μL Lipofectamine 2000 was mixed with 100 μL OPTI-MEM. After 5 minutes of incubation at room temperature, the two were mixed by gentle pipetting and incubated for 20 to 30 minutes at room temperature to allow siRNA/lipid complexes to form. At the end of incubation, the 200 μL transfection solution was used to resuspend the cell pellet (~1.5 × 10^5^ cells/well). After incubation at room temperature for 10 minutes, regular growth medium was added at a ratio of 1:5 (1 volume of transfection solution/4 volumes of growth medium) and the cell suspension was transferred to the culture plate. After 24 hours of incubation at 37°C in 5% CO_2_, the medium was replaced with fresh growth medium. RNAs and proteins were extracted and analyzed at the indicated time points after transfection. Plasmid DNA transfections were carried out as described for siRNA, except that 1 μg DNA in 25 μL OPTI-MEM and 1 μL Lipofectamine 2000 in 25 μL OPTI-MEM were used for each well of cells (the 50 μL of final transfection reagent with 500 μL of regular growth medium was added to each well).

### In vivo SAHH activity assay.

The experiments were performed in a 96-well scale using the Human Homocysteine (Hcy) ELISA Kit (MBS260128, Mybiosource) that allows quantitative measurement of homocysteine concentration in cell extracts, according to the manufacturer’s instructions. The concentration of homocysteine in cell extracts was used as a readout for in vivo SAHH activity because SAHH hydrolyzes SAH to homocysteine and adenosine. Briefly, cells were washed with cold PBS and lysed on plates in 200 μL of lysis buffer (40 mM hexadecyltrimethylammonium bromide, 75 mM Tris-HCl, 1 M NaCl, 15 mM EDTA). The lysate was cleared of insoluble materials by centrifugation at 15000*g* at 4°C for 15 minutes. Immediately after the centrifugation, 100 μL of the supernatant was collected and used for SAHH activity measurement. The absorbance of the samples was determined using a Multi-Mode Microplate Reader (Fluoroskan Ascent FL Microplate Fluorometer and Luminometer, Thermo Fisher Scientific).

### Antibodies, siRNAs, and plasmids.

See Supplemental Methods for details.

### Western blot and quantitative RT-PCR.

See Supplemental Methods for details.

### RRBS and methylation-specific PCR.

See Supplemental Methods for details.

### Microscopy and DTI experiments.

See Supplemental Methods for details.

### Statistics.

See Supplemental Methods for details.

### Study approval.

All present studies in animals were reviewed and approved by an appropriate institutional review board and approved by the Institutional Animal Care and Use Committee of Yale University (IACUC 2013-10614).

## Author contributions

MM, EB, MP, AB, and DS designed experiments. MS, CB, SO, LZ, DC, FH, AS, VH, and MM performed and analyzed experiments. KI performed the statistical analyses. MM, AB, and MS wrote the manuscript. All data are available in the main text or the supplemental materials.

## Supplementary Material

Supplemental data

Supplemental data set 1

## Version 1 10/22/2021

Electronic publication
