# Supplementary material for: Preimplantation factor modulates oligodendrocytes by H19-induced demethylation of NCOR2
Source: JCI Insight. 2021 Oct 22;6(20):e132335. doi: 10.1172/jci.insight.132335 (PMC8564895; doi:10.1172/jci.insight.132335)
Supplement: Supplemental data [file jciinsight-6-132335-s028.pdf]

Supplementary Materials

PreImplantation Factor modulates oligodendrocytes by H19 induced de-methylation of NCOR2

Marialuigia Spinelli[1], Celiné Boucard[1], Sara Ornaghi[2], Andreina Schoeberlein[1], Keller Irene[3], Daniel Coman[4], Fahmeed Hyder[5], Longbo Zhang[6], Valérie Haesler[1], Angelique Bordey[6], Eytan Barnea[7], Michael Paidas[2]; Daniel Surbek[1], Martin Mueller[1,2*]

Correspondence to: martin.mueller@insel.ch

This PDF file includes:

Materials and Methods
Fig. S1 and 2
Table S1

Materials and Methods

Antibodies, siRNAs, and plasmids:

We used following antibodies and concentrations for staining: anti-OLIG2 alexa fluor 488 conjugate (Merck MABN50A4, 1:100); anti-CNPase alexa fluor 488 conjugate (Biocompare bs-1000R-A488, 1:50); anti-MASH1 (BD Pharmingen 556604, 1:200), and anti-MBP (Bioss bs-0380R-A488 1:200). Where appropriate, we used the secondary antibody alexa fluor goat anti-rabbit 488 (Abcam 150077, 1:200). For western blots we used following antibodies and concentrations: rabbit antibody against NCOR2 (Abcam, ab24551, 1:400), mouse monoclonal anti-b-actin (Sigma, A2228, 1:100), CNPase (Abcam, ab227218, 1:500), rabbit antibody against Olig-2 (Abcam, ab109186, 1:300), rabbit antibody against MBP (Chemicon International, AB980, 1:500). siRNAs specific for human *H19* (siH19, 4390816/n253566), and negative siCon (AM4636) were purchased from Ambion/Life Technologies. Plasmid expressing human *H19* (pH19) and empty vector were used as previously described (*1*). Plasmid expressing human NCOR2 was purchased from Santa Cruz biotechnology (sc-401150 SMRTe CRISPR/Cas9 KO Plasmid (h)). Synthetic PIF (MVRIKPGSANKPSDD) and scrambled PIF (scrPIF: MVRIKEGSANKPSDD) was provided by Bio-Synthesis, Inc. (Lewisville, TX, USA). Peptide identity was verified by matrix-assisted laser desorption/ionization time-off light (MALDI-TOF) mass spectrometry and amino-acid analysis, and the peptides were purified to >95% by HPLC.
To make pCMV-H19^{CA} plasmid that expresses full-length H19 (pH19), PCR was carried out using human term placental cDNA as a template and forward 52-aaggaaaaaagcggccgcAGCAGGGTGAGGGAGGGGGTG and reverse 52-cgcggatccGTAACAGTGTTTATTGATGATGAGTC primers (the lower-case letters represent restriction enzyme sites for NotI and BamHI, respectively, and the upper-case letters are complementary to H19). The resulting 2663-bp long PCR fragment was ligated to pFLAG-CMV-2 (Sigma) opened with NotI and BamHI. Effects induced by this plasmid are noted as H19^{CA} induced.

Protein Isolation and Western Blotting

Protein levels were determined by SDS polyacrylamide gel electrophoresis followed by blotting against their specific antibodies as previously described (*2-4*). All western blotting experiments were performed in triplicates.

RNA and genomic DNA extraction and Real-Time polymerase chain reaction

RNA was extracted using the QIAshredder and the Allprep DNA/RNA/Protein Mini Kits (Qiagen, Hilden, Germany) according to the manufacturer's protocol. Genomic DNA was isolated using Quick-gDNA MicroPrep (D3021, Zymo, Germany) according to the manufacturer's instructions (*2-4*). The quality and concentration of RNA and DNA was assessed by a NanoVue Plus™ spectrophotometer (Biochrom). Up to 5 µg of total RNA were reverse transcribed by the SuperScript III First-Strand Synthesis System (Thermo Fisher Scientific, US). Real-time quantitative PCR was performed in a 12 µl reaction containing 1 µl of the eluate using TaqMan Gene ® Expression Master Mix, (4369016, Thermo Fisher Scientific, US). Gene expression levels were normalized against house-keeping gene GAPDH. Following primers were used:

Mbp (Forward: 5'-ACTATCTCTTCCTCCCAGCTTAAAAA-3'; reverse: 5'-TCCGACTATAAATCGGCTCACA-3')

Cnpase (Forward: 5’-GCCGCCGGGACATCA-3’; reverse: 5’-ACTGGTCGGCCATTTCAAAG-3’)

Claudin11 (Forward: 5’-CTGGCTGGTGTTTTGCTCATT-3’; reverse: 5’-CGCACACAGGGAACCAGAT-3’)

Olig2 (Olig2, GE assay ID: Hs00300164_m1, Thermo Fisher Scientific, US)

H19 (H19, GE assay ID: Hs00399294_m1, Thermo Fisher Scientific, US)

Smrt (*Ncor2*, GE assay ID: Hs00196955_m1, Thermo Fisher Scientific, US)

Gapdh (Forward: 5’-GCTCCTCCTGTTCGACAGTCA-3’; reverse: 5’-ACCTTCCCCATGGTGTCTGA-3’)

<u>Reduced Representation Bisulfite Sequencing (RRBS) and Methylation-specific PCR (MSP)</u>

Genomic DNA was extracted from cells using Quick-gDNA MicroPrep (Qiagen, Hilden, Germany). For bisulfite treatment, 400-500ng of DNA was used for each column using EZ DNA Methylation-Gold Kit (D5006, Zymo, Germany). DNA Methylation Profiling was performed by Diagenode (Diagenode Cat# G02020000) as previously reported (*2*). RRBS libraries were sequenced generating 50 bp single reads on an Illumina® HiSeq 4000 instrument. The sequenced reads were controlled for quality. Adapter removal was performed using Trim Galore! version 0.4.5_dev. The cleaned reads were then aligned to the Homo sapiens reference genome (Genome Reference Consortium Human Build 37, GRCh27, hg19) using Bismark v0.16.1. Once the percentage of methylation was computed, the reported cytosines were filtered to get only the CpGs covered. The spike-in control sequences were used at this step to check the bisulfite conversion rates and to validate the efficiency of the bisulfite treatment. Quality control and adapter trimming for all samples was carried out using "Trim Galore!" software. The default Phred score of 20 was used as the quality cut-off value. For trimming the required overlap with adapter sequence (stringency) parameter was set to a value of “1”, while maximum error rate was defined as “0.1”. Minimum read length was defined as 15bp. Quality control of data was undertaken using “FastQC” software. Calculation of DNA methylation levels and per base methylation calls were performed subsequently in the “Bismark” pipeline based on the human reference genome hg19. Further quality checks and tests for differential methylation (DM) were performed with MethylKit v. 1.4.0 in R v. 3.4.2. We retained only CpG positions covered by at least 3 reads and not in the top 0.1% in terms of coverage. Coverage was normalised among samples. Pairwise comparisons between experimental groups were run for all CpGs covered in at least 2 samples per group using logistic regression and a correction for overdispersion. Models were compared with Chi-square tests (*5*). CpGs were considered DM if they had SLIM adjusted P-values below 1% and a minimum methylation difference of 25% (*6*). DMs were annotated to human Entrez genes and CpG islands using the Bioconductor package annotatr v. 1.4.0.

For MSP, QMSP was performed as previously described (*2*) (*7*). Briefly, PCR was performed in a 12 µl reaction containing 1 µl of the eluate using TaqMan Gene ® Expression Master Mix, (4369016, Thermo Fisher Scientific, US). Following primers were used: *Ncor2* 218372R Lot 1726393 - A10 - Taqman SNP genotyping Assay. This is a custom made primer by Thermo Fisher Scientific, US that contain the "methyl" mutation at the predicted position on the reverse DNA strand. The PCR primers for methylated DNA were used at a final concentration of 0.3 mM in each PCR reaction. PCR was performed by initial denaturation at 95°C for 5min, followed by 40 cycles of 30 s at 95°C, 30 s at 60°C and 30 s at 72°C. Specificity was verified using melting curve

analysis and agarose gel electrophoresis. The Ct values of each sample were used in the post-PCR data analysis. Albumin DNA was used as loading controls for all QMSP normalization.

Microscopy and Cell Counts

We performed cardiac perfusion with PBS followed by formaldehyde (4%; Merck, Whitehouse Station, NJ) prior to brain harvesting. Brains were removed surgically and fixed in formaldehyde solution (4%) for 2-4 hours at room temperature (RT) followed by 4°C for a total time of 24-48 hours. Additionally, tissue for fluorescence immunohistochemistry were cryoprotected with 30% sucrose in PBS, and stored at –20 °C in 30% glycerol, 30% ethylene glycol until used. Coronal cryo-sections (6 µm) were obtained with a sliding freezing microtome (Leica SM2000R) and incubated with target antibodies following manufacture`s instruction. Briefly, antigen retrieval was performed on cryo-sections with a pressure cooker in a Na-citrate pH6 solution. After 12 minutes the pressure cooker was cooled down in a cold bath during 20 minutes. The slides were transferred to a sequenza slide rack (Coverplate Technology). TBS-T was pipet between the slides and the sequenza cover slide to check the capillary gap and used after each antibody as wash solution. Serum block (10% goat serum, 1% BSA and 1% Triton100-x) was applied during 2 hours at RT. After 3 washing with TBS-T we used the primary antibodies and incubated overnight at 4°C. The sections were washed again 3 times and counterstain with DAPI. In case of indirect immunohistochemistry the secondary antibody was incubated during 1 hour at RT after the first antibody and washing steps. All images were acquired with BX51 microscope (Olympus, Tokyo, Japan) or Leica DM6000B microscope (Leica Microsystems, Wetzlar, Germany) using a 40× objective and equipped with a digital camera. An independent observer blinded to the experimental conditions acquired images per animal, condition, and each specific immunostaining. At P3, we analyzed the labeled cells from the SVZ in coronal sections of region of interest (ROI) using high-low settings to minimize saturation. We reconstructed images and manually counted for the cell marker (Mash-1) using ImageJ 1.39t software. We used DAPI routinely as a counterstain to count cell nuclei and calculate the percentage of SVZ cells expressing a specific marker. At P3 we defined ROI as three to four sections containing the dorsal SVZ. At P7 we defined ROI as six consecutive coronal sections containing corpus callosum and deep cortical layers as the injury induced at P0/1 in mice corresponds to human immature infants in terms of brain development and expected injury site (*8*, *9*). Each specific immunostaining was analyzed in the labeled cells resulting in percentage of SVZ cells expressing the specific marker. Additionally, we measured MBP intensity (*10*). Briefly, intensity was analyzed in four regions of the corpus callosum per section at three sections per brain. Measurement involved acquiring color images at the same exposure level, converting images to 8-bit gray scale (fluorescence intensity from 0 to 255), and calculating mean intensity (compared to contralateral hemisphere) in the region from pixels above the threshold excluding background fluorescence.

DTI experiments

The DTI datasets were obtained on a 9.4 T horizontal bore magnet (Bruker, Billerica, MA, USA) with a custom-made ^{1}H radio frequency (RF) coil. The DTI experiments were performed using the Stejskal-Tanner spin-echo diffusion-weighted sequence with a diffusion gradient of 5ms and a delay between the two diffusion gradients of 15ms. 24 coronal slices of 0.5mm thickness were acquired using a repetition time (TR) of 2s and an echo time (TE) of 25.1ms and 16 averages. Two Shinnar–Le Roux (SLR) pulses of 1ms each were used for excitation and inversion, respectively. The images were acquired at 128 × 64 resolution and reconstructed to 256 × 256, resulting in an in-plane resolution of 100 µm × 100 µm. 15 diffusion-weighted images were acquired,

corresponding to noncollinear diffusion directions with the same b=1000 s/mm^2 and 1 with no diffusion weighting. The diffusion tensor was calculated for each voxel from the intensities of the 15 diffusion-weighted images as previously described (*11*) (*12*). The tensor eigenvalues (λ_1, λ_2 and λ_3) and the corresponding eigenvectors were obtained by matrix diagonalization. The fractional anisotropy (FA) was calculated using the following equation in the defined ROI:

$$FA = \sqrt{\frac{3}{2}\frac{(\lambda_1 - \lambda_{avg})^2 + (\lambda_2 - \lambda_{avg})^2 + (\lambda_3 - \lambda_{avg})^2}{\lambda_1^2 + \lambda_2^2 + \lambda_3^2}}$$

where $\lambda_{avg} = (\lambda_1 + \lambda_2 + \lambda_3)/3$. The FA images were generated using BioImage Suite (http://www.bioimagesuite.org/).

Statistical analysis

All quantifications such as manual cell counts were performed in a blinded manner. Positive cell counts were performed in the ROI (see above) as previously reported (*4*). Number of cells was determined by unbiased counting of positive cells (*4*). We avoided variability resulting from tissue handling and staining, and inter-animal developmental variations by using a ratio of left (ipsilateral to carotid occlusion) to the right hemisphere (*4*). All data are represented as mean±S.E.M. Single comparisons with control were made using two-tailed Student’s t-test or Mann–Whitney test with Bonferroni’s correction if necessary. We used one-way repeated measures ANOVA followed by Bonferroni’s Multiple Comparison Test for multigroup design. $P<0.05$ was considered to be statistically significant. Data handling and statistical processing were performed using the Microsoft Excel and GraphPad Prism Software.

Study approval

All present studies in animals were reviewed and approved by an appropriate institutional review board and approved by the Yale University, New Haven, USA - Institutional Animal Care and Use Committee (IACUC# 2013-10614).

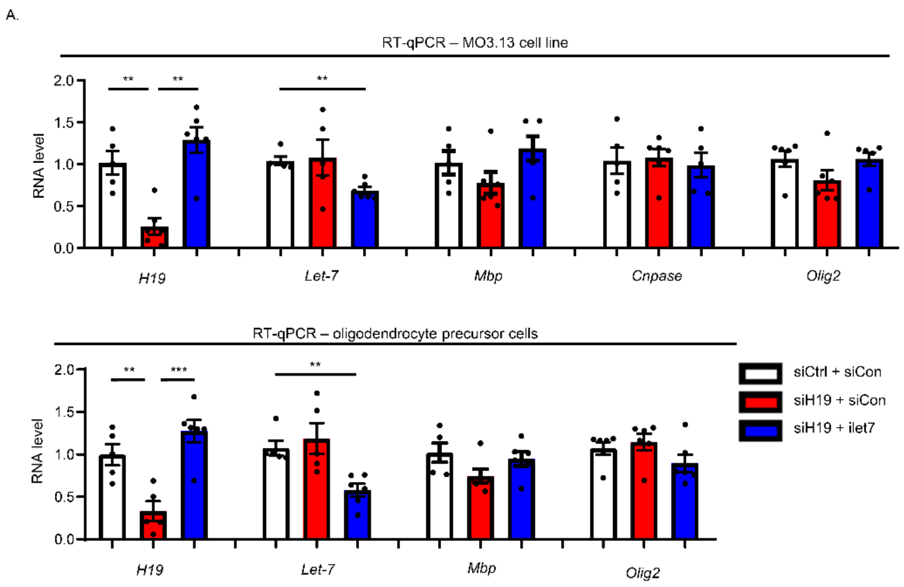

Fig. S1. sPIF induces oligodendrocyte differentiation markers in H19 dependent manner.

Hybrid cell line (MO3.13) that expresses phenotypic characteristics of primary oligodendrocytes in an immature developmental stage and rat oligodendrocyte precursor cells were transfected with control siRNA (siCtrl) and/or control miRNA (siCon) and siRNA specific for H19 (siH19) or let-7 inhibitor (iLet7) and RNAs were harvested 48h post-transfection and analyzed by qRT-PCR. Results are presented as mean $\pm$ S.E.M. Single comparisons to control were made using two-tailed Student's t-test or Mann-Whitney test with Bonferroni correction. **p<0.005; ***p<0.0005. The levels of Ctrl and siCtrl were arbitrary set as 1. Protein levels are presented after normalization against Actin. Each experiment was conducted at least three times.

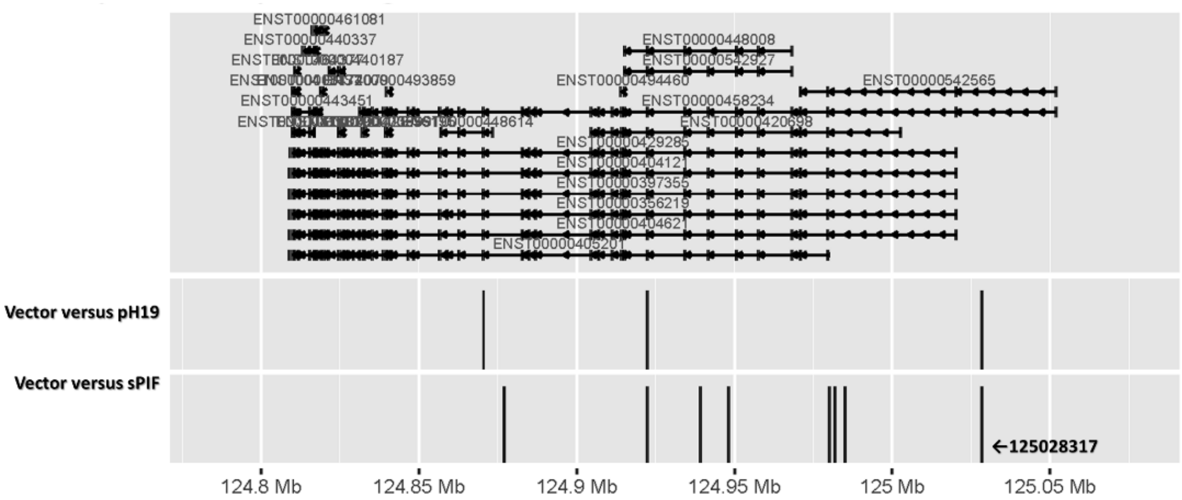

Fig. S2. ***Ncor2*** **CpG island methylation changes**

A plot showing differentially methylated CpGs using Ensembl genome browser of *Ncor2*. pH19 and sPIF hypomethylated the same intron region of *Ncor2* (marked with the sequence mark position). Numbers below indicate the identified positions of the indicated nucleotides in the chromosome.

Table S1.

RNA-seq and methylation profile data of cells treated with Vector, pH19, and sPIF (n=3 each group).

References:

1. S. Ghazal *et al.*, H19 lncRNA alters stromal cell growth via IGF signaling in the endometrium of women with endometriosis. *EMBO molecular medicine* **7**, 996-1003 (2015).
2. J. Zhou *et al.*, H19 lncRNA alters DNA methylation genome wide by regulating S-adenosylhomocysteine hydrolase. *Nature communications* **6**, 10221 (2015).
3. M. Mueller *et al.*, PreImplantation factor promotes neuroprotection by targeting microRNA let-7. *Proceedings of the National Academy of Sciences of the United States of America* **111**, 13882-13887 (2014).
4. M. Mueller *et al.*, PreImplantation Factor bolsters neuroprotection via modulating Protein Kinase A and Protein Kinase C signaling. *Cell death and differentiation* **22**, 2078-2086 (2015).
5. K. Wreczycka *et al.*, Strategies for analyzing bisulfite sequencing data. *Journal of biotechnology* **261**, 105-115 (2017).
6. H. Q. Wang, L. K. Tuominen, C. J. Tsai, SLIM: a sliding linear model for estimating the proportion of true null hypotheses in datasets with dependence structures. *Bioinformatics* **27**, 225-231 (2011).
7. J. Deng *et al.*, H19 lncRNA alters methylation and expression of Hnf4alpha in the liver of metformin-exposed fetuses. *Cell death & disease* **8**, e3175 (2017).
8. L. S. Hsieh *et al.*, Convulsive seizures from experimental focal cortical dysplasia occur independently of cell misplacement. *Nature communications* **7**, 11753 (2016).
9. N. Salmaso, B. Jablonska, J. Scafidi, F. M. Vaccarino, V. Gallo, Neurobiology of premature brain injury. *Nature neuroscience* **17**, 341-346 (2014).
10. B. Oppliger *et al.*, Intranasal Delivery of Umbilical Cord-Derived Mesenchymal Stem Cells Preserves Myelination in Perinatal Brain Damage. *Stem cells and development* **25**, 1234-1242 (2016).
11. K. A. Park *et al.*, Excitatory Synaptic Drive and Feedforward Inhibition in the Hippocampal CA3 Circuit Are Regulated by SynCAM 1. *The Journal of neuroscience : the official journal of the Society for Neuroscience* **36**, 7464-7475 (2016).
12. Y. Jung *et al.*, An epigenetic mechanism mediates developmental nicotine effects on neuronal structure and behavior. *Nature neuroscience* **19**, 905-914 (2016).
